# A Collaborative Approach to Address Racism in a Community–Academic Partnership

**DOI:** 10.5888/pcd20.220365

**Published:** 2023-06-08

**Authors:** Erin Lebow-Skelley, Martha Scott Tomlinson, Simone Charles, Christina Fuller, Bren Ames, Melanie A. Pearson

**Affiliations:** 1 Emory University, Rollins School of Public Health, Gangarosa Department of Environmental Health, HERCULES Exposome Research Center, Atlanta, Georgia; 2 University of Michigan, School of Public Health, Environmental Sciences, Ann Arbor, Michigan; 3 HERCULES Exposome Research Center, Stakeholder Advisory Board, Atlanta, Georgia; 4 University of Georgia, College of Engineering, School of Environmental, Civil, Agricultural and Mechanical (ECAM) Engineering, Athens, Georgia; 5 Aye Open Outcomes, National Human Ecology Action League (HEAL), Atlanta, Georgia

## Abstract

The HERCULES Exposome Research Center at Emory University uses an exposome approach to study the environment’s effect on health and community well-being. HERCULES is guided by a Stakeholder Advisory Board (SAB) that includes representatives of neighborhoods, nonprofit organizations, government agencies, and academic institutions in the Atlanta metropolitan region. This region (and the SAB) has a large proportion of Black residents, many of whom live in areas experiencing environmental injustices. Historic and current racial injustices in Atlanta and public health research made it imperative to initiate dialogue and implement actions to address racism and power dynamics that may impact research and partnerships between affected communities and our institution.

After initial discussion, the HERCULES Community Engagement Core and SAB members formed a workgroup to develop an internal anti-racism process. The workgroup drafted an Anti-Racism Commitment, hosted a Racism and Equity Dialogue Series, and initiated a strategic planning process to implement the resulting recommendations, which fell into the following categories: anti-racist guidance/policies and recommendations for research, community engagement, and the department. Center leadership and the SAB were engaged throughout the iterative process.

This deliberate and ongoing process allows HERCULES to identify and begin implementing action items that go beyond a written proclamation to address racialized power imbalances and systemic inequities. HERCULES is committed to working collaboratively to earn community trust while addressing systemic issues, recognizing that these are essential to forming research partnerships that address health inequities.

**Summary**

**What is already known on this topic?**

Racism has permeated public health research and academia, and academic research centers and partner communities play an important role in creating more equitable outcomes for all those involved in research endeavors.

**What is added by this report?**

Our report describes a collaborative process that academic research centers and community partners may adapt to address institutional racism and embed anti-racism, equity, and justice into their operations and structures.

**What are the implications for public health practice?**

Creating antiracist research structures, collaboratively with community partners, has the potential to promote health equity and improve research relevance, translation, and impact. This report highlights a strategy for others to transform their practice toward meaningful change.

## Background

The American Public Health Association named racism as a public health crisis in 2020 (1). To address racism in a lasting way, public health research needs to be viewed through a critical lens that strengthens and promotes racial equity. Racism has long permeated science and public health research (2). For example, Black and American Indian communities have been the subjects of research, often without their knowing consent, such as the US Syphilis Study at Tuskegee and the genetic research among the Havasupai Tribe (3,4). Historically, research institutions have engaged with communities in an extractive manner, taking knowledge and data and giving little in return (5,6). Academic institutions, including Emory University, have begun to acknowledge their long history of racism (7), including barriers to entry for racial and ethnic minority groups (8). There is also a need to increase the number of scholars of color (9,10) and incorporate anti-racism into the institution (11), the curricula (12), and, specifically, into environmental health science and community-engaged research (8,9,13). In response to a renewed national awakening to racialized injustices, the HERCULES Exposome Research Center’s (HERCULES) Community Engagement Core (CEC) and Stakeholder Advisory Board (SAB) initiated a process to identify and address racism in HERCULES’ practices.

**Figure 1:**
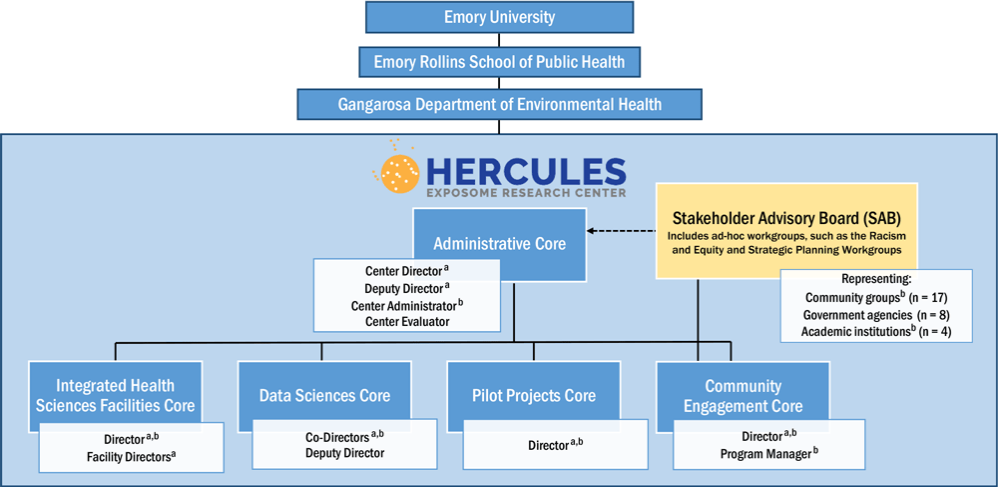
HERCULES organizational chart. Footnote a indicates members of the HERCULES Leadership team. Footnote b indicates members of the Center Anti-Racism Workgroup.

HERCULES is an environmental health research center at Emory University funded by the National Institute of Environmental Health Sciences to support exposome research, with the goal of capturing the totality of environmental exposures across the lifespan to better understand the environment’s contribution to health and disease, including chronic disease (14–16). Situated within the Rollins School of Public Health, HERCULES has 77 members who are faculty from across the school and university; one-third are Environmental Health faculty. The Center helps its members incorporate the exposome into their research by providing support in data science, targeted and untargeted chemical analysis, pilot project funding, and community engagement (Figure 1). Community engagement is integral to many federally funded research centers, often with dedicated cores like the HERCULES CEC (17–19). The HERCULES CEC has built a long-term and committed relationship with its SAB, both formed as part of the Center in 2011, with several original members still serving today. The active 29-member SAB includes representatives of community groups and organizations (n = 17), government agencies (n = 8), and other academic institutions (n = 4) who are focused on environmental health and justice issues in the Atlanta metropolitan region. Community members are compensated for their time, knowledge, and unique perspectives. The SAB oversees and provides community perspectives to CEC activities, offers connections to the local community, and provides critical guidance to HERCULES toward fulfilling its mission to improve exposome science and environmental health and justice in the Atlanta metropolitan region.

The Atlanta metropolitan region is one of the largest metropolitan areas in the southeastern US, including 11 counties and over 6 million people. Most residents are people of color (56%), predominantly Black residents (33%), which is more than double the proportion of the US Black population (12%) (20,21), and HERCULES SAB members reflect this diversity. This diverse region faces myriad environmental injustices that impact residents’ health. The region has the nation's largest racial wealth gap (22), ranks near last in upward intergenerational mobility (23), and has outdated infrastructure, including a combined stormwater/sewer system that contributes to excess flooding and sewer overflows (24–27). Atlanta’s pollution sources are predominantly located in areas with a large population of color (28,29) that also face high levels of poverty, limited access to healthy foods and transportation, and higher rates of asthma and breast cancer (28–32), resulting in part from racist policies like redlining (32,33), highway placement, transit boundaries, and urban renewal projects that continue to displace, fragment, and isolate Black neighborhoods, maintaining Atlanta’s historic segregation (34–36).

Racism has been linked to chronic disease outcomes (32,37–39), and some have posited that the exposome concept should include exposures such as racism (40,41). Also, Emory University and HERCULES researchers are predominantly White, and Emory University has a history of slavery and dispossession (42), reinforcing the need to address systemic and institutional racism (10). A 2015 focus group with SAB members indicated that community-based SAB members lacked trust in Emory, expressed distrust of the university’s motives, perceived it to be a school for the privileged, and believed that research results are not always communicated back to communities. However, the same focus group indicated high levels of trust for CEC staff due to their reliability and follow-through. As such, it was imperative for the CEC to maintain that trust by initiating dialogue and implementing actions to address racism and power dynamics that could harm our partnerships, research, and impact. Academic research centers like HERCULES have the potential to affect faculty, students, and the surrounding community negatively or positively, by either continuing extractive research practices or engaging in collaborative, anti-racist research that pursues racial and health equity. The long history of racism cannot be overcome by passive means, but must instead be directed by anti-racist practices.

Anti-racism is the active practice of identifying and opposing racism and supporting policies that reduce racial inequity (43). While others have noted the need to incorporate anti-racism into academic curricula (12), anti-racist practices must be incorporated beyond the classroom and throughout the institution and, when possible, should be developed with the involvement of community partners. Academic research centers need to adopt anti-racist practices as a prerequisite to create more equitable research and power sharing for all those involved in research endeavors (44). HERCULES and its SAB have initiated this anti-racist transformation in the HERCULES program, and we share our process here so that others may apply it in their efforts to dismantle racism in their institutions and partnerships.

## The HERCULES Anti-Racism Process: From Dialogue to Action

**Figure 2:**
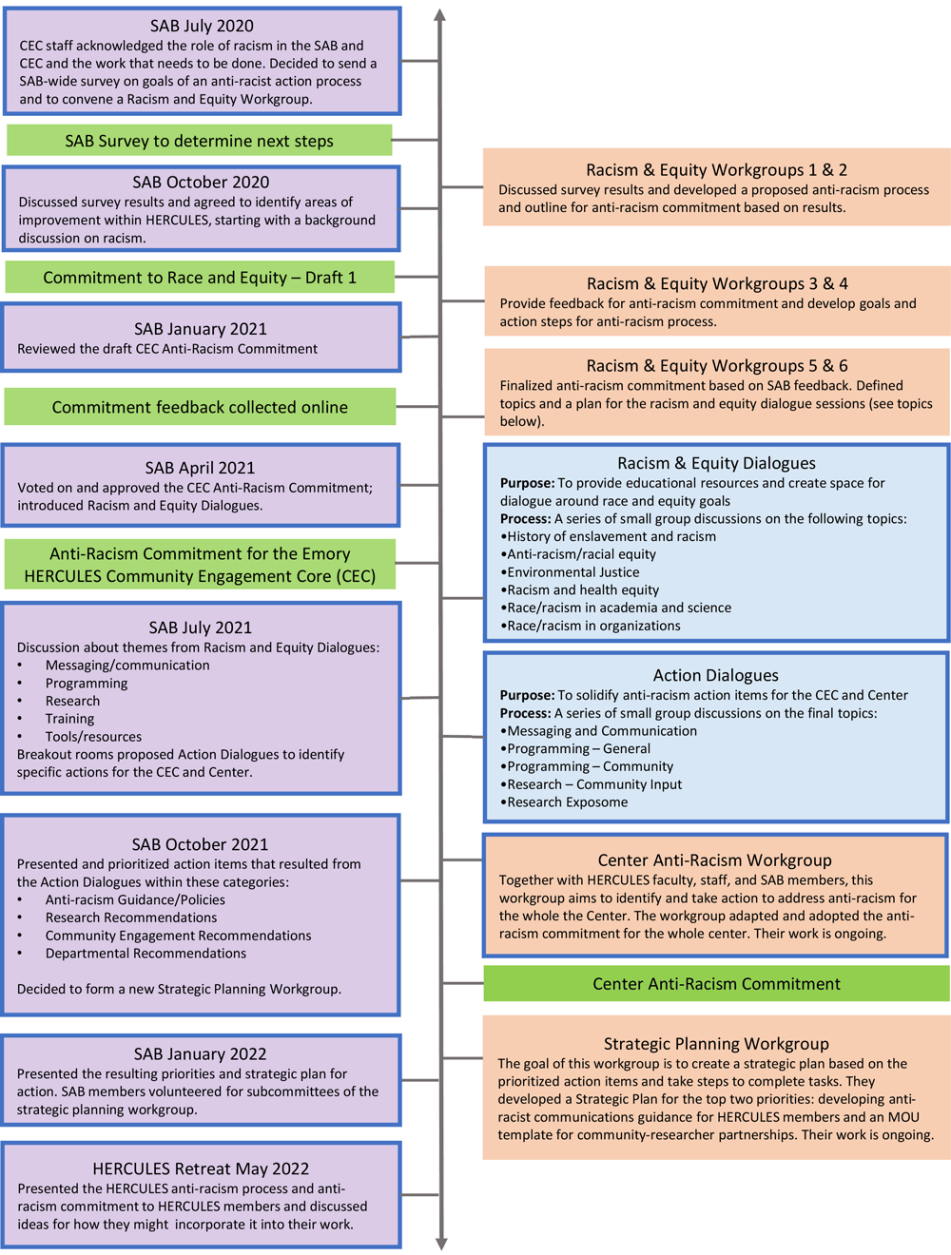
HERCULES pathway to addressing racism and equity. Purple boxes indicate a HERCULES Meeting (SAB or Retreat). Peach boxes indicate a Work Group, blue boxes indicate a Dialogue Session, and green boxes indicate an Output. Blue outlines indicate participation from HERCULES leadership and/or faculty. Abbreviations: CEC, Community Engagement Core; MOU, memorandum of understanding; SAB, Stakeholder Advisory Board.

The SAB and CEC initiated an ongoing anti-racism process within HERCULES in July 2020. We describe this process in detail, with the timeline depicted in Figure 2.

### Initial SAB input and survey

The first step was to acknowledge the role of racism within HERCULES, the CEC, and the SAB and determine whether and how the SAB wanted to move forward with an anti-racism process. To do this, we initiated the discussion at a quarterly SAB meeting, which led to the recommendation that we hold dedicated discussions about racism and equity, possibly with a facilitator. We followed up with a survey to the SAB to determine next steps, including how these discussions should be structured, the topics (anti-racism broadly, institutional racism, and/or bias within HERCULES), and who should be included in these discussions (n = 21, 72% response rate). Discussion topic ratings were closely ranked, the top being to discuss racial and ethnic bias within HERCULES (n = 11). Respondents described their goals for the discussions, with many wanting to improve HERCULES processes and operations, and to include background and education around racism, anti-racism, and institutional bias specific to public health. Most SAB members felt that these initial discussions should include the full SAB (n = 15, 71%), CEC staff (n = 17, 81%), and HERCULES leadership (Center Director [n = 15, 71%] and core leaders [range, 9–13, 43%–62% across core leads]). To complete these steps, 4 SAB members volunteered to form the Racism and Equity Workgroup to provide additional guidance on this effort. During the initial workgroup meetings, we discussed the survey results, brainstormed ideas for an anti-racism process, and drafted language and values to include in an anti-racism commitment.

### Anti-Racism Commitment for the CEC

Building from the language and values emphasized by workgroup members, the CEC staff developed a first draft of the CEC’s Anti-Racism Commitment. During the following 6 months we received and incorporated feedback through an iterative process between CEC staff, the workgroup, and the SAB. The final Commitment was approved by the full SAB during a quarterly meeting.

### Racism and equity dialogues

Using the SAB survey results, the workgroup decided the dialogues’ goal was to identify areas of improvement within HERCULES policies and operations and to provide background and education of the history and systems of racism so that all participants (SAB members and HERCULES leadership) worked from a shared foundation, context, and language. Meanwhile, outside facilitators were in high demand, with limited availability and high fees. As such, the workgroup decided to host educational discussions internally, with SAB members and HERCULES leadership volunteering to facilitate. The idea of a book club approach emerged at an SAB meeting: small group discussions, with a list of resources around a certain topic.

The workgroup further refined plans for the dialogues to spark discussions around racism and equity while providing a foundation to identify priorities to address within HERCULES. Reflecting on the survey results and HERCULES’ mission, the workgroup used a brainstorming process to identify 6 main topics that provided a historical background on racism and covered how racism specifically impacts community partnerships, public health, and research (Figure 2). Workgroup members and CEC staff gathered resources (eg, news articles, journal articles, videos, podcasts, presentations) for each topic in a shared online document. SAB members and HERCULES leadership were invited and registered for dialogue sessions. Some topics had more than one session due to the level of interest, and individuals could sign up for as many sessions as they wanted.

Nine sessions of virtual dialogues were conducted and attended by 28 people (including 5 members of HERCULES leadership); 8 were facilitated by SAB members, one by a member of HERCULES leadership. Per feedback received on the SAB input survey and because each session was only an hour, sessions were limited to 8 participants, not including CEC staff, to make sure there was time for every participant’s voice to be heard. Before each session, participants were asked to read, listen to, or watch at least 1 resource from the discussion topic resource list and to come prepared to discuss it. Volunteer facilitators guided sessions using a facilitation guide co-developed by CEC staff and the workgroup, and a CEC staff member attended to take notes and participate when appropriate. As conversations about racism and discrimination can be psychologically demanding, each session started with an introduction activity to help participants pause to think about how they were feeling and consider their intention for participating in the conversation. We provided participants with a feelings wheel (45) and asked them to share 1 to 2 words for 1) how they were feeling and 2) their intention for the session, along with their name and organization. We then reviewed our 9 community agreements (46–48) to help establish ground rules and create an inclusive and respectful space for the conversation. Next, each person provided a brief overview and key takeaway of the resource they reviewed (eg, “What was something you found the most interesting or didn’t know before?”). The group then discussed “How does this relate to something you have seen or experienced with the SAB/CEC/HERCULES?” and determined recommendations and takeaways to report back to the SAB (eg, “We want HERCULES to know or consider this. . . .”). These dialogues provided a space for HERCULES leadership and SAB members to talk together about these tough issues while generating rich ideas for areas of improvement and change within the SAB, CEC, and HERCULES to dismantle racism and promote equity.

### Action dialogues

Following the dialogues, CEC staff reviewed the notes, compiled a list of recommendations, and identified 5 common themes (Figure 2). The time from starting the process through presenting the common themes was 1 year. During the meeting, we asked members to self-select into breakout groups to identify specific action items regarding communication, programming, and research. (The other topics were specific recommendations that didn’t require further discussion.) In each topic-specific breakout group, SAB members agreed on which recommendations they wanted to implement and brainstormed next steps, including who would do it, how, and when. After the breakout discussions, the SAB decided we needed more time to answer these questions and recommended that we schedule another round of dialogues: action dialogues.

The purpose of the action dialogues was to solidify action items associated with specific topics (Figure 2). CEC staff helped facilitate these discussions, with a member of HERCULES leadership participating in each to ensure that they were aware of and involved in the recommended actions.

CEC staff reviewed all the notes from the action dialogues and identified 4 main categories with 4 or 5 specific recommendations each. Primary categories included anti-racism guidance/policies, research recommendations, community engagement recommendations, and departmental recommendations. We presented these action items at an SAB meeting, where SAB members rated each action on a 5-point scale from lowest to highest priority (n = 19) (Table 1). Four SAB members volunteered for a strategic planning workgroup to move these priorities forward.

**Table 1:** HERCULES Stakeholder Advisory Board Anti-Racism Action Items, by Category and Ranking^a^

Action item	Priority ranking
**Anti-racism guidance/policies**	
Messaging/communication guidance for HERCULES members’ publications, presentations, etc	1st
MOU template for community–researcher partnerships	1st
Guidance document for scientists to use when developing research projects	2nd
Develop and implement training on all guidance	2nd
**Research recommendations**	
Incorporating race/racism into exposome research methods	1st
Support HERCULES junior scientists of color	2nd
Provide ongoing training for HERCULES researchers about anti-racism and community engagement	2nd
Recommendations specific to HERCULES Pilot Program	4th
**Community engagement recommendations**	
Showcase the work of community grantees to facilitate collective action and networking with researchers and local communities	2nd
Create an advocacy training program for community grantees	3rd
Implement structures to ensure student projects follow anti-racism and best practices in community engagement	4th
Coordinate an Atlanta-wide, community-engaged research ethics forum	5th
Design multifaceted youth engagement program	5th
**Departmental recommendations**	
Re-examine faculty promotion and tenure	3rd

Abbreviation: MOU, memorandum of understanding.

a This table is meant to serve as an example of the action items that resulted from our year-long process. Each action item is a brief description of the detailed discussions and decisions that were made.

## Ongoing Work

### Strategic Planning Workgroup

The Strategic Planning Workgroup developed a plan to implement the top 2 priority recommendations: 1) developing anti-racist messaging/communication guidance and 2) creating a standard memorandum of understanding (MOU) for community–researcher partnerships (Table 1). The third highest priority item, to incorporate racism into exposome science, was referred to the Center Anti-Racism Workgroup.

### Center Anti-Racism Workgroup

After participating in the CEC’s dialogue series, HERCULES leadership determined that they needed to form a Center-level workgroup to implement some of the SAB recommendations and also identify other actions required at the Center level. Center leadership and SAB members comprise the Center Anti-Racism Workgroup (Figure 1). Its first task was to modify the CEC’s Anti-Racism Commitment to apply to the whole Center. Although Center Workgroup members agreed that the CEC Commitment could largely be adopted as-is by the full Center, they identified areas to expand to include the full purview of the Center, such as its influence over Center-level recruitment and mentorship and its members, faculty representing all departments within the School of Public Health and many across the university. The finalized HERCULES Anti-Racism Commitment is posted on the HERCULES website (49) and was shared at the 2022 HERCULES Retreat, with a discussion between SAB members and HERCULES faculty about how to apply the commitment in their work.

The Center workgroup is now working on a recommendation that emerged from both the action dialogues and the retreat discussion: to host training/seminars for faculty and others to learn about how to incorporate race and racism into exposome science.

## Evaluation

We have monitoring mechanisms built into this process to track our work and report on progress and accomplishments at our quarterly SAB meetings and in funder progress reports. Here, we report on a process evaluation assessing the implementation and short-term outcomes of this ongoing collaborative process. Using document review and a mixed-methods participant survey co-designed by our SAB member co-authors, we report on the initiative’s implementation and participation, accomplishments and short-term outcomes, and participants’ attitudes and satisfaction with the process (50,51). Together, these provide a basis for assessing the strengths and weaknesses of the process thus far.

### Accomplishments

To date, the process has produced several tangible outcomes. First, the Anti-Racism Commitment guides HERCULES in its mission to improve environmental health in the metropolitan Atlanta area. A major part of the commitment is to build equity into all procedures, programs, and activities, such as our purchasing and procurement practices, publications and publishing practices, funding criteria, and evaluation activities. For example, we intentionally purchased event supplies from a local Black-owned business (March 2022) and amplified scholars of color within our citations in a publication (April 2022) (10,52,53), just 2 actions to share power.

Second, HERCULES leadership has demonstrated its continued investment throughout this process. They participated in the dialogue sessions, provided feedback for the CEC Anti-Racism Commitment, adapted and adopted it for the full Center, and established a Center Anti-Racism Workgroup.

Third, our process created a living list of specific, prioritized action items for the CEC and Center to address, being carried forward by the Strategic Planning Workgroup and the Center Anti-Racism Workgroup (Table 1). This work reaches beyond the Center given that HERCULES members represent all departments in the School of Public Health, including the Chair of the Gangarosa Department of Environmental Health. Additionally, the HERCULES director serves as the Executive Associate Dean for Faculty Affairs and Research Strategy and 3 core leads serve on their department’s Diversity, Equity and Inclusion Committee.

### Participant attitudes

We solicited feedback about the HERCULES anti-racism process from the SAB and leadership via a survey that inquired about their perceptions, satisfaction, and concerns. Twenty-four people responded to the survey, 19 SAB members (65.5%) and 5 members of HERCULES leadership (62.5%). Respondents rated the importance of 5 process activities (Table 2). Overall, 90% of respondents felt that the activities were important or very important to the process. One SAB Workgroup member wrote that the process was “the best I had participated in compared with other[s] that did it too quickly and in less depth and commitment.” SAB members rated the SAB/Center Workgroups and the Anti-Racism Commitment most important (100% and 92%, respectively). Center leadership unanimously rated 3 of the 5 activities as “important” or “very important.” All respondents from the HERCULES leadership team and about 90% of SAB respondents felt that the process had been a good use of their time and CEC staff time (Table 3). Seventy-nine percent of respondents stated that the process was either successful or very successful, while 21% chose neutral (Table 3).

**Table 2:** Activity Participation and Importance Rating From HERCULES Stakeholder Advisory Board and Leadership (N = 24)

Activity	No. of participants in activity	Participant rating, no. (%)^a^					No. of missing participants
		Not important	Less important	Neutral	Important	Very important	
**HERCULES stakeholder advisory board members (n = 19, response rate 65.5%)**							
Anti-racism discussions at SAB meetings	13	0	1 (7)	2 (13)	2 (13)	10 (67)	4
Workgroup (SAB or center)	9	0	0	0	4 (33)	8 (67)	7
Small group dialogue sessions	11	0	0	2 (15)	4 (31)	7 (54)	6
Anti-racism commitment	9	0	1 (8)	0	2 (17)	9 (75)	7
Anti-racism discussion at HERCULES retreat	7	0	1 (8)	1 (8)	1 (8)	9 (75)	7
**HERCULES leadership (n = 5, response rate 62.5%)**							
Anti-racism discussions at SAB meetings	3	0	0	0	1 (25)	3 (75)	1
Workgroup (SAB or center)	3	0	0	0	1 (25)	3 (75)	1
Small group dialogue sessions	4	0	0	0	2 (40)	3 (60)	0
Anti-racism commitment	4	0	1 (20)	0	0	4 (80)	0
Anti-racism discussion at HERCULES retreat	3	0	0	1 (20)	1 (20)	3 (60)	0

Abbreviation: SAB, stakeholder advisory board.

a Denominator for % in each column is the total number of respondents for that question, not activity participation. Total number of respondents is calculated by summing the number of responses for “not important,” “less important,” “neutral,” “important,” and “very important” categories.

**Table 3:** Overall Reflection from HERCULES Stakeholder Advisory Board and Leadership (N = 24)

Reflection question	Strongly disagree, n (%)^a^	Disagree, n (%)	Neutral, n (%)	Agree, n (%)	Strongly agree, n (%)
Has this process been a valuable use of your time?					
SAB	0	0	2 (10)	4 (21)	13 (68)
Leadership	0	0	0	2 (40)	3 (60)
Has this process been a valuable use of our staff time?					
SAB	0	0	2 (10)	3 (16)	14 (74)
Leadership	0	0	0	1 (20)	4 (80)
**Reflection question**	**Not at all successful, n (%)^b^**	**Slightly successful, n (%)**	**Neutral, n (%)**	**Successful, n (%)**	**Very successful, n (%)**
How successful do you think this process has been at addressing race and racism in HERCULES and the work we do?					
SAB	0	0	4 (21)	8 (42)	7 (37)
Leadership	0	0	1 (20)	2 (40)	2 (40)

Abbreviation: SAB, stakeholder advisory board.

a Total number of respondents is calculated by adding up the number of responses for “strongly disagree,” “disagree,” “neutral,” “agree,” and “strongly agree” categories.

b Denominator for % in each column is the total number of respondents, not participants. Total number of respondents is calculated by adding up the number of responses for “not at all successful,” “slightly successful,” “neutral,” “successful,” and “very successful” categories.

The survey also asked respondents about their concerns. A common theme that emerged was the need to implement the identified action items and the necessity to continue work in the area. One SAB member wrote, “Too early to [assess] whether the process has been successful to address racism as that is a longer-term goal, but this is definitely a huge step in the right direction.” Another concern was about the amount of information and complexity of the topic. For example, one SAB Workgroup member wrote, “I was feeling overloaded with information and not much time to process during most calls. However, HERCULES staff helped with that . . . there were many recaps and HERCULES staff could repeat, slow down, go over material upon request.” These survey results helped us reflect on the strengths and weaknesses of this process.

### Strengths and weaknesses

The HERCULES anti-racism process has several strengths. It is a collaborative, in-depth, iterative, transparent process. Steps evolve with input from SAB members, Center leadership, and CEC staff. Everyone serves as an equal partner in the process, and this model of self-guided learning and sharing was a cost-effective, socially distanced option, given the COVID-19 pandemic and the high demand for facilitators. The process encourages active engagement of all participants, enlisting community and academic members alike as learners and educators to share resources, observations, ideas, and recommendations (54). Active engagement throughout the process results in greater buy-in and potentially more immediate implementation, further facilitated by the inclusion of Center leadership in all stages of the process. This full inclusion also means everyone is aware of and begins to practice what we commit to doing collectively, so translation and implementation are more certain to permeate and guide processes and procedures (55,56).

One of the long-term goals of the process is to inform and create change in the Center’s operations which could transcend to university and community operations. Having intentional action as a metric of success increases the possibility of embedded changes (54). In addition, this process, built for members by members, remains deliberate and ongoing, with continuous feedback allowing participants to engage in various ways according to their comfort levels, desired level of engagement, and knowledge about racism and anti-racism, building trust and commitment between participants, in the work itself, and the Center’s direction (57).

The process also has challenges. It started over 2 years ago, proceeds slowly, and has no end point, which could result in attrition of participants over time. However, CEC staff regularly provide an overview of past activities and progress when needed. Another challenge could result from HERCULES leadership being fully engaged in every stage of the work, with power differentials potentially adversely influencing how transparently participants engage (56). However, the CEC is guided by community-based participatory principles (5) where power dynamics are considered and intentionally mitigated to reduce this effect. To this end, the initial survey let the SAB guide the development of the process, asking if they wanted Center leadership to participate. The follow-up survey did not indicate any resulting concerns from leadership’s participation.

## Conclusion

The structure, history, and trust within the HERCULES SAB as well as the nationwide attention being given to the topic has enabled us to embark on a process to address systemic racism within our institution and partnerships. We acknowledge the institutionalized barriers that exist, including that HERCULES is part of a predominantly White institution in a city with a large proportion of Black residents and historical and ongoing environmental injustices. The ongoing, iterative work to become an anti-racist, multicultural organization must be grounded in trust, earning community willingness to develop mutually beneficial, long-term collaborative partnerships and then co-planning and implementing an intentional, transparent process together as respected partners and colleagues. The anti-racism process described here can serve as a roadmap for others in their efforts to dismantle racism within their institutions and partnerships.
